# Supplementary material for: Development, Validation, and Application of High-Performance Liquid Chromatography with Diode-Array Detection Method for Simultaneous Determination of Ginkgolic Acids and Ginkgols in Ginkgo biloba
Source: Foods. 2024 Apr 19;13(8):1250. doi: 10.3390/foods13081250 (PMC11049217; doi:10.3390/foods13081250)
Supplement: Supplementary file 1 [file foods-13-01250-s001.zip › foods-2928589-supplementary.pdf]

Supplementary Material

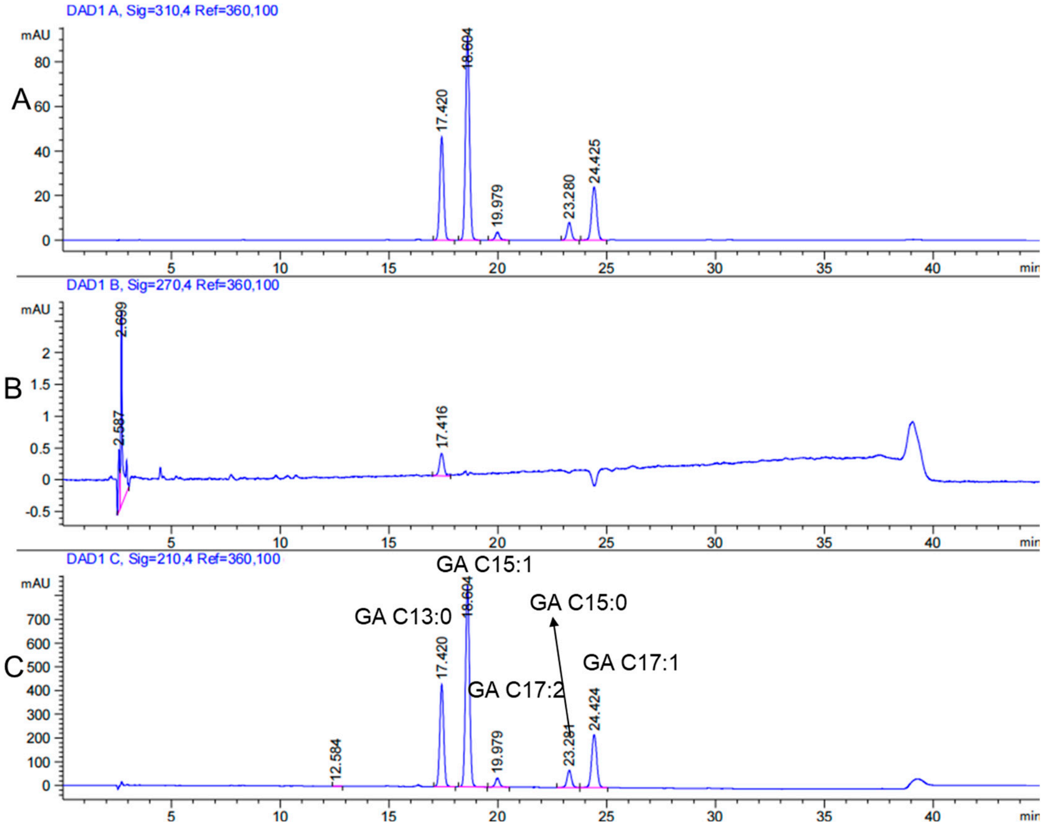

Figure S1. Chromatogram of ginkgolic acid mixed standard at 310nm (A), 270 nm (B) and 210 nm (C) by the European Pharmacopoeia methods.

Table S1. Linear regression equation, correlation coefficient, linear range, and LOD of ginkgols.

Ginkgol	Standard curve	R^2	Linear range (ppm)	Limit of detection (ppm)	Limit of quantification (ppm)
C13:0	y = 15785x + 3.10	0.9998	0.35-56.56	0.61	2.01
C15:1	y = 11592x + 2.81	0.9999	0.27-53.00	0.50	1.65
C17:1	y = 8699.9x + 0.57	0.9999	1.39-69.40	0.06	0.20

Table S2. Coefficients of the eigenvectors were taken from different principal components analysis for compounds.

Compound	PC1	PC2	PC3
GA C13:0	0.4222	-0.01345	-0.04245
GA C15:1	0.34351	0.41681	0.19673
GA C17:1	0.35682	0.38546	0.16816
Total GA	0.36034	0.3757	0.16568
G C13:0	-0.14599	0.51813	-0.83165
G C15:1	-0.36905	0.34138	0.16371
G C17:1	-0.38126	0.25864	0.33343
Total ginkgols	-0.37909	0.29064	0.27231

Table S3. Pearson correlation showing the relationship between ginkgolic acids and ginkgols.

Compound	GA C13:0	GA C15:1	GA C17:1	Total GA	G C13:0	G C15:1	G C17:1	Total ginkgols
GA C13:0	1							
GA C15:1	0.79504							
GA C17:1	0.82825	0.99835						
Total GA	0.8369	0.99735	0.99988					
G C13:0	-0.33806	0.02387	-0.00352	-0.01451				
G C15:1	-0.87849	-0.43103	-0.48003	-0.49352	0.54769			
G C17:1	-0.91643	-0.49691	-0.54585	-0.55849	0.40163	0.97416		
Total ginkgols	-0.90895	-0.47551	-0.5249	-0.53795	0.45838	0.98759	0.99738	1
